# Oral health-related quality of life in Bangladeshi children of sex workers: socio-behavioural and oral health predictors

**DOI:** 10.1186/s12955-025-02381-z

**Published:** 2025-05-13

**Authors:** Afia Mahmuda Khan, Taseef Hasan Farook, Sumaiya Zabin Eusufzai, Mehnaj Sharmin, Sabrin Shohid, Tabassum Zerin, Lameea Shahed, Sheikh Jamal Hossain, Mohammad Delwer Hossain Hawlader

**Affiliations:** 1 Department of Public Health, North South University, Dhaka, 1229 Bangladesh; 2 NSU Global Health Institute (NGHI), North South University, Dhaka, 1229 Bangladesh; 3 Public Health Promotion and Development Society (PPDS), Dhaka, 1205 Bangladesh; 4 Maternal and Child Health Division (MCHD), International Centre for Diarrheal Disease Research, icddr,b, Mohakhali, Dhaka, 1212 Bangladesh; 5 Adelaide Dental School, University of Adelaide, Adelaide, SA 5000 Australia; 6 School of Dental Sciences, University Sains Malaysia, 16150 Kelantan, Malaysia; 7 Department of Public Health and Hospital Administration, National Institute of Preventive and Social Medicine (NIPSOM), Dhaka, 1212 Bangladesh; 8 Global Health and Migration Unit, Department of Women’s and Children’s Health, Uppsala University, Uppsala, Sweden

**Keywords:** Female sex workers, Oral health-related quality of life, Caries, Child perception questionnaire, Oral impact on daily performances

## Abstract

**Background:**

Children of female sex workers (FSWs) in Bangladesh grow up in a challenging socio-economic environment characterized by parental separation, substance abuse, alcoholism, and limited access to healthcare, including oral health services. This study aimed to assess the oral health-related quality of life (OHRQoL) and its associative factors among these children.

**Materials and methods:**

A cross-sectional study was conducted between March 2023 and February 2024 with a sample of 180 FSW mothers/institutional caregivers and their school-going children, aged 7 to 17. OHRQoL was assessed using the CPQ_8 − 10_ (Child Perception Questionnaire) for children aged 7 to 11, and the OIDP (Oral Impacts on Daily Performances) for adolescents aged 12 to 17. The Decayed, Missing and Filled Teeth (DMFT/dmft) index was used to assess dental caries, while the gingival index evaluated gingival health. The plaque index and calculus index were employed to assess plaque and calculus levels, respectively.

**Results:**

Among children aged 7 to 11, oral symptoms (Mean = 5.36, SD = 3.72) and functional limitations (Mean = 4.57, SD = 4.42) were the most affected parameters. For children aged 12 to 17, 78.2% reported oral impacts on their daily performances (Mean = 6.50, SD = 6.84). Caries status (β = 0.361, *p* = 0.001) and place of residence (β = 0.329, *p* = 0.032) were significantly associated with higher CPQ_8 − 10_ scores. A higher OIDP score was associated with gingivitis (β = 0.265, *p* = 0.035).

**Conclusion:**

The study reveals that children aged 7 to 11 in Daulatdia with caries had significantly poorer OHRQoL, while gingivitis was associated with a higher OIDP score in children aged 12 to 17. OHRQoL evaluation is vital in determining the effectiveness of therapeutic and preventive measures aimed at improving the oral health of this vulnerable population.

**Registry and registration no. of the study:**

The Institutional Review Board /Ethics Review Committee (IRB/ERC) of North South University reviewed and approved this study(2023/OR-NSU/IRB/0204).

## Background

The term “prostitutes” or “sex workers” refers to women who engage in sexual activities, either regularly or irregularly, for legal or illicit purposes, in exchange for payment or other forms of economic compensation [1]. According to the Bangladesh Immoral Activities Control Act of 1933, commonly known as the Prostitution Act, a woman over the age of eighteen may voluntarily engage in prostitution as long as she adheres to legal requirements. However, within Bangladesh’s societal framework, commercial sex work is highly stigmatized and operates in unfavourable social conditions [2, 3]. As of now, the estimated number of female sex workers (FSWs) across various typologies and age groups in Bangladesh stands at 109,624, with a majority being over the age of 25 [4]. Many of these women are drawn into sex work through internal human trafficking [5]. FSWs are often labelled as “high-risk” individuals, “vectors of disease,” or victims of coercion. Such characterizations fail to acknowledge the diverse and complex experiences of these women [6]. Poverty, lack of education, and social stigma create a precarious situation for both sex workers and their children [7, 8].

Children born into brothels or associated with the sex industry are often seen as “tainted” by societal standards. This stigma leads to deep feelings of shame, regardless of whether they are directly involved in the sex industry or simply associated with it [9]. These children grow up in challenging environments marked by parental separation, substance abuse, alcoholism, and limited access to healthcare and education. Insufficient supervision often results in them following career paths they were exposed to in their formative years [10–12]. Due to the taboo surrounding prostitution, the children of FSWs are frequently neglected, leaving them marginalized and vulnerable. This neglect impacts both their physical and psychological development, further entrenching the cycle of disadvantage [13, 14].

Dental disorders are more prevalent in underprivileged communities, contributing to both oral health inequalities and broader socioeconomic disparities [15]. Dental caries, the most common chronic disease in children and adolescents, represents the greatest global oral health burden despite being largely preventable [16]. The link between oral health issues and overall health, encompassing physical, social, and psychological well-being, is well-established [17]. Consequently, there is an increasing clinical focus on improving quality of life as an essential goal of dental care [18, 19]. This has led to the development of socio-dental indicators, such as oral health-related quality of life (OHRQoL) measures [20, 21] which pertains to the impact of oral conditions on an individual’s daily functioning, overall health and quality of life.

Children suffering from dental pain, abscesses, gum disease, and damaged teeth may exhibit signs of distress, which can negatively affect their social, functional, and psychological well-being. These conditions may also increase the risk of hospitalization, leading to higher treatment costs [22]. However, few studies have examined the social, educational, and health outcomes of children of FSWs in Bangladesh, and none have focused specifically on their oral health [7, 23]. To address this gap in the literature, our research aimed to evaluate the oral health-related quality of life and its predictors among children of FSWs.

## Material and method

### Study design and study setting

From March 2023 to February 2024, a cross-sectional study was conducted to assess the OHRQoL among primary and high school-aged children of FSWs. To carry out this research, two nonprofit organizations (NPOs) in Dhaka City—Shishuder Jonno Amra (SJA) and KK Foundation (KKF)—as well as one daycare center, BASHA, and Mukti Mohila Samity (MMS), a nongovernmental organization (NGO) near the Daulatdia Brothel, were selected using convenience sampling.

BASHA rescues sex workers and their children from brothels across Bangladesh while offering income-generating opportunities to the mothers. Similarly, SJA and KKF provide residential, educational, and medical care to the children of sex workers. MMS, a women’s and children’s rights organisation was established as part of the ALO (Alternative Livelihood Opportunity) initiative and focuses on child development programs for children of sex workers at Daulatdia Brothel.

Due to the lack of available documentation, convenience sampling was necessary to obtain the sample. Dental camps were organized at the four study locations at 15-day intervals, with FSWs, institutional caregivers, and their children (ages 7 to 17) attending. An institutional caregiver is a volunteer or employee of an institution who is in charge of a child’s welfare, whether they receive payment or not [24]. In this study, Institutional caregivers are those who are associated with the NGO and have caregiving responsibilities for the children of sex workers. To maximize recruitment, participation was open to all camp attendees. However, due to various circumstances, many potential participants were unavailable. As a result, a first-come, first-enrolled approach was adopted to finalize the sample.

### Participants

The sample size was calculated using maternal oral health knowledge of their children in Bangladesh. The calculation employed a standard error of 7%, a 95% confidence interval (CI), and a prevalence of 18.5% [25] of mother’s oral health awareness about their 5 to 9-year-old children in Dhaka. The primary sample size estimation was 157. The sample size was determined to be 188 individuals after accounting for the nonresponse rate of 20%. However, after compensating for missing values and screening the data, the final sample size was determined to be 180. Because of accessibility constraints, the sample size was kept small. Details of the sample size calculation are provided in ‘Supplementary file 1’. With the use of a convenient sampling technique, data was gathered from the four study areas: 50 samples were obtained from the MMS Center, 66 samples from SJA, 9 samples from KKF, and 63 samples from BASHA. The study included women who were sex workers or institutional caretakers, together with their children aged 7 to 17 years who did not have any physical or mental health difficulties. Primary education is intended for students aged 7 to 11, while secondary school education is for those aged 12 to 17, according to UNICEF [26].

### Data collection

OHRQoL related information was obtained from each children of FSWs through face-to-face interviews. sociodemographic variables as well as oral health-related information and the quality of life of their children. Data collection was conducted in the four study locations.

Four dentists performed oral clinical examinations on children, and two of them calibrated their assessments using the World Health Organization’s (WHO) standard approach. The children were examined in an organization or childcare setting and sat on chairs with the examiner in front of them. The teeth were dried with cotton pellets, and a torchlight and natural sunshine were employed for visibility before oral health assessment. The WHO recommends that scoring caries be done using mouth mirrors and CPI ball-ended probes as examination tools [27].

### Research tool

A structured questionnaire was utilized to evaluate the sociodemographic trait, OHRQoL oral health status.

- Part A of the questionnaire gathered sociodemographic information, including the child’s age, sex, education, the mother’s or caregiver’s age, education, and whether the child resided with the mother or caregiver.
- Part B consisted of Child Perception Questionnaire (CPQ_8-10)_ index for children aged 7 to 11 years and Oral Impacts on Daily Performances (OIDP) scale for children aged 12 to 17 years.

### Oral health-related quality of life

Oral health-related quality of life of children of sex workers was measured using CPQ_8 − 10_ and OIDP questionnaires. The CPQ_8 − 10_ questionnaire, validated in low resource setting, consisted of 25 questions and four subscales. In this questionnaire, oral symptoms in the first subscale (5 questions), functional limitations in the second subscale (5 questions), emotional well-being in the third part (5 questions), and social well-being in the fourth part (10 questions) were questioned [28]. The frequency of the events in the previous four weeks concerning the child’s oral/orofacial condition was evaluated for all questions. The responses were scored as a five-point Likert scale (never: 0, once/twice: 1, sometimes: 2, often: 3, every day/almost every day: 4). A total score and subscale scores were calculated by summing up all scores; the higher score pointed higher effect on the quality of life, indicating worse child OHQoL. Jokovic et al. [28] used CPQ_8 − 10_ for 8 to 10 age group. However, we used this questionnaire for the age group of 7 to 11 years old.

An expert panel comprising 5 professionals (2 from public health, 1 from social sciences, and 2 from dentistry) reviewed and provided feedback on the adopted questionnaire, which was then revised accordingly. The questionnaire was translated into Bangla from English, then re-translated into English, and subsequently translated back into Bangla. This forward and backward translation process was carried out by two language experts fluent in both Bangla and English.

A pilot study involving 50 mothers with school-going children aged 7 to 11 years was conducted to pretest the translated CPQ_8 − 10_ questionnaire. Internal consistency reliability among each item of the scale was tested by analyzing Cronbach’s alpha. This was 0.91 for total CPQ_8 − 10_ scale, indicating an excellent level of internal consistency. For overall CPQ_8 − 10_ scale, the corrected item-total correlation coefficients ranged from 0.34 to 0.70 for the total CPQ_8 − 10_ scale. The coefficients for the subscales varied from 0.45, which was indicative of lower coefficients for the oral symptom’s domain, to 0.73 for the emotional well-being domain.

When the OIDP inventory was first developed, adults were the target audience [17] and subsequently adapted for children [29]. The OIDP questionnaire was validated in Bangladeshi adolescents and adult population [30]. Oral impacts on daily performances were obtained by adding scores for eight frequency items. “During the past six months how often have problems with your mouth and teeth caused you any difficulties with, 1) eating, 2) speaking and pronouncing clearly, 3) cleaning teeth, 4) sleeping and relaxing, 5) smiling without embarrassment, 6) maintaining emotional state, 7) enjoying contact with other people and 8) carrying out major school work. The scale used was in the following range:

(0) “never affected”,
(1) “less than once a month”,
(2) “once or twice a month”,
(3) ”once or twice a week”.
(4) “3–4 times a week”,
(5) “every or nearly every day”.

The range of possible total OIDP scores is 0 to 40. Poorer OHRQoL was associated with higher OIDP scores.

### Clinical assessment

Dental caries was assessed in accordance with WHO 2013 guidelines to ascertain decayed, missing, and filled teeth (DMFT) for permanent tooth indices and decayed, missing, and filled teeth (dmft) for deciduous teeth [31]. To determine the numerical expression of caries prevalence, the total values of dmft and DMFT were computed both separately and jointly as the sum of d + m + f + D + M + F. After assigning scores, the level of dental caries severity was established by determining whether dmft + DMFT = 0 (caries-free) or dmft + DMFT > 0 (caries present) [32]. In 1963, Silness and Löe established the gingival index (GI), which was used to assess each person’s gingival health state [32], and dental plaque was measured using the Silness and Löe plaque index (1964) in the study sample [33, 34]. Mineralized deposits were graded using Ramfjord’s (1959) calculus index approach [35]. In order to collect data for DMFT, plastic chairs, artificial light, blunt edge caries probes, and disposable plastic mirrors were used. In designated dental camps across four research regions, the children’s oral health status was evaluated. Six dental clinicians were trained and calibrated in the assessment of oral health status prior to the commencement of these dental camps. The consistency of the oral health status examination was determined using intra- and inter-examiner reliability tests. The intra- and inter-examiner reliability was evaluated using Cohen’s Kappa statistic, which yielded values of 0.84 and 0.86, respectively.

### Statistical analysis

Statistical analyses were carried out using IBM SPSS version 26. A frequency distribution test was used to explain socio-demographic and oral health status and behavior-related variables. Independent sample t-tests were performed to compare the CPQ_8 − 10_ and OIDP scores between socio-demographic and oral health status and behaviors-related variables, with the level of significance set to 5% (*p* < 0.05). Multiple linear regression analysis was applied to determine the relationship between the dependent (CPQ_8 − 10_ and OIDP total scores) and independent variables. The independent variables in this regression included socio-demographic characteristics such as gender of children (0 = Female,1 = Male), age (0 = 18–30 years 1 = > 31 years) and education (0 = Primary or less,1 = Secondary or higher) of mothers/ institutional caregivers, type of caregivers (0 = FSW Mother,1 = Institutional Caregiver), place of residence (0 = Daulatdia,1 = Dhaka), oral health status such as caries (0 = Absent,1 = Present), plaque (0 = Absent,1 = Present), calculus (0 = Absent,1 = Present) and gingivitis (0 = Absent,1 = Present).

### Ethical approval and informed consent

The study was conducted following the Declaration of Helsinki. The Institutional Review Board/Ethics Review Committee (IRB/ERC) of North South University, Dhaka, Bangladesh (2023/OR-NSU/IRB/0204) provided ethical clearance. Prior to the interview, each participant was thoroughly briefed about the study’s objectives and methodologies. The mothers who could read and write signed the informed consent forms. Mothers provided written consent prior to the oral assessment and data collection.

The mothers gave their written consent prior to the oral evaluation and data collection. They received informed consent papers outlining the goals and objectives of the study one day prior to data collection. Literate mothers signed the forms independently, whereas those who were not literate had an individual capable of reading and writing read the forms to them prior to signing.

Children ages 7 to 11 gave verbal consent, while those ages 12 to 17 gave written informed consent on the following page of the consent letter from their mothers. The children’s caregivers provided data for the mothers who were unable to participate. The mothers or caregivers signed consent forms prior to the survey. The research only included female sex workers or caregivers who had given consent, as well as their offspring.

## Results

The children aged 7 to 17 were examined with respect to socio-demographics (Table 1**).** Out of 180 participants, a noteworthy percentage (58.9%) were female. According to the age distribution of the children, 56.7% of children were found to be between the ages of 7–11 years. A majority of FSW’s children (72.2%) resided in Dhaka city. On the other hand, the Daulatdea brothel housed a quarter (27.8%) of the children. Approximately 41% of the mothers/caregivers were between the ages of 18 and 30. Furthermore, about 33.3% of FSW mothers had completed secondary or higher education, compared to 66.7% of mothers who had only completed elementary school. Of the respondents, 54% were mothers and 46% were institutional caregivers.

**Table 1:** Frequency distribution of sociodemographic characteristics of children of FSWs

Variables	Socio-Demographic Information	
	*N*	%
**Gender of children**		
Female	106	58.9
Male	74	41.1
**Age of children**		
7–11 years	102	56.7
12–17 years	78	43.3
**Age of mothers/caregivers**		
18–30 years	74	41
>31 years	106	59
**Education of children**		
Primary School	102	56.7
High School	78	43.3
**Education of mothers/caregivers**		
Primary or less	120	66.7
Secondary or higher	60	33.3
**Type of caregivers**		
FSW Mother	97	54
Institutional Caregiver	83	46
**Place of residence**		
Daulatdia	50	27.8
Dhaka	130	72.2

In terms of data pertaining to oral health (Table 2), more than half of the children surveyed (52.8%) had dental decay, which is indicative of poor oral hygiene. Most children (71%) had visible plaque. About 62.8% of children had signs of calculus. On the other hand, 65% of the children in the study, showed no evidence of gingivitis, indicating that their gingival health was generally in good condition. Regarding self-reported oral health-related behaviour, 55.6% of the children brushed their teeth twice/more a day and 94% of the children used toothpaste. Over half (50.6%) used to rinse their mouth after meals/sweets/fizzy drinks.

**Table 2:** Frequency distribution of oral health-related information of children of FSWs

Variables	Oral Health Related Information	
	*N*	%
**Caries**		
Caries Free	85	47.2
Caries Present	95	52.8
**Plaque**		
Absent	52	29
Present	128	71
**Calculus**		
Absent	67	37.2
Present	113	62.8
**Gingivitis**		
Absent	117	65
Present	63	35
**Frequency of daily tooth brushing**		
Once	80	44.4
Twice or more	100	55.6
**Which dentifrice does your child use for brushing his or her teeth**		
Toothpaste	169	94
Toothpowder	11	6
**Rinsing the mouth after meals/sweets/fizzy drinks**		
Yes	89	50.6
No	89	49.4
**Cleaning tongue regularly**		
Yes	58	32.2
No	120	66.7

Table 3 shows the measures of central tendency and variability of the scores obtained of the students in each dimension of CPQ_8 − 10_. For children aged 8–10 years, the most affected dimensions were oral symptoms 5.36 (SD 3.72) and functional limitations 4.57 (SD 4.42). The mean total CPQ_8 − 10_ score was 16.92 (SD 13.87).

**Table 3:** Measures of central tendency and variability of the scores obtained of the students in each dimension of the CPQ_8 − 10_

Dimension	CPQ_8 − 10_ Total Score			
	Mean(SD)	Median	Min	Max
**Oral symptoms**	5.36(3.72)	5.00	0.00	16.00
**Functional limitations**	4.57(4.42)	4.00	0.00	17.00
**Emotional well-being**	3.26(3.95)	2.00	0.00	20.00
**Social well-being**	3.72(5.46)	0.00	0.00	20.00
**Total score**	16.92(13.87)	15.00	0.00	61.00

SD: Standard deviation

Table 4 reveals the prevalence, central tendency, and variability of oral impact on daily performances among the 12-17-year-old children of FSW. The overall prevalence of oral impact was 78.2%. In terms of daily performances, the highest prevalence of oral impact was reported on eating (60.3%) and cleaning teeth (60.3%), followed by sleeping (30.8%) and emotional stability (27%). Oral impacts on studying (14%) and social contact (14%) were reported to be among the lowest. The mean OIDP total score was 6.50 (SD 6.84). The mean OIDP score for each performance, from the highest to the lowest, were related to eating (1.86, SD 1.78), cleaning teeth (1.55, SD 1.53), sleeping (0.79, SD 1.38), emotional stability (0.63, SD 1.19), speaking (0.54, SD 1.11), smiling (0.47, SD 1.09), social contact (0.33, SD 0.907) and studying (0.32, SD 0.904).

**Table 4:** Prevalence, central tendency and variability of oral impact on daily performances among the 12- to 17-year-olds children of FSWs

Oral Impacts	Prevalence(%)	Mean(SD)	Median	Min	Max
**Overall Impact**	78.2	6.50(6.84)	4.5	0	31
**Eating**	60.3	1.86(1.78)	2	0	5
**Speaking**	23	0.54(1.11)	0	0	5
**Cleaning Teeth**	60.3	1.55(1.53)	1.5	0	5
**Sleeping**	30.8	0.79(1.38)	0	0	5
**Smiling**	19.2	0.47(1.09)	0	0	5
**Emotion**	27	0.63(1.19)	0	0	5
**Study**	14	0.32(0.904)	0	0	4
**Social Contact**	14	0.33(0.907)	0	0	4

SD: Standard deviation

Table 5 illustrates the association of socio-demographic characteristics, oral health status, and oral health-related behaviour with CPQ_8 − 10_ and OIDP score. Children aged 7 to 11(CPQ_8 − 10_) living in Daulatdia (*p* value = 0.015) and experiencing dental caries (*p* value < 0.001) found to have significantly poorer OHRQoL compared to their counterparts. Additionally, children aged 12 to 17 who exhibited signs of gingivitis (*p* value = 0.033) had a significantly higher overall OIDP score compared to those without gingivitis. No association was found between oral health-related behaviour with CPQ_8 − 10_ and OIDP score.

**Table 5:** Association of sociodemographic characteristics, oral health status and oral health related behaviours with CPQ_8 − 10_ and OIDP scores

Variables	CPQ_8 − 10_ Total Score			OIDP Total score		
	Mean ± SD	*p* Value	*t* value	Mean ± SD	*p* Value	*t* value
**Gender of children**						
Female	16.77 ± 14.26	0.911	-0.111	6.06 ± 6.32	0.446	-0.769
Male	17.08 ± 13.59			7.44 ± 7.87		
**Age of mothers/caregivers**						
18–30 years	17.05 ± 13.92	0.917	0.104	6.53 ± 4.79	0.981	0.024
> 31 years	16.77 ± 13.97			6.49 ± 7.41		
**Education of mothers/caregivers**						
Primary or less	17.17 ± 13.06	0.823	0.225	6.75 ± 7.25	0.549	0.604
Secondary or higher	16.51 ± 15.27			5.81 ± 5.65		
**Type of caregivers**						
FSW Mother	19.14 ± 12.327	0.072	-1.82	7.92 ± 7.43	0.066	-1.868
Institutional Caregiver	14 ± 15.34			5.08 ± 5.94		
**Place of residence**						
Daulatdia	20.62 ± 12.98	**0.015**	-2.452	5.60 ± 3.65	0.617	0.527
Dhaka	14.00 ± 13.96			6.56 ± 7.01		
**Caries**						
Caries Present	20.53 ± 13.81	**< 0.001**	-4.286	6.59 ± 5.09	0.923	-0.098
Caries Free	9.71 ± 11.02			6.45 ± 7.64		
**Plaque**						
Present	17.16 ± 13.51	0.829	-0.221	6.17 ± 6.71	0.41	0.843
Absent	16.53 ± 14.64			8.00 ± 7.48		
**Calculus**						
Present	17.32 ± 14.17	0.764	-0.301	6.43 ± 6.81	0.88	0.152
Absent	16.49 ± 13.68			6.72 ± 7.12		
**Gingivitis**						
Present	20.97 ± 15.36	0.079	-1.795	8.48 ± 7.46	**0.033**	-2.18
Absent	15.24 ± 12.95			5.04 ± 6.02		
**Frequency of daily tooth brushing**						
Once	17.19 ± 12.61	0.842	0.200	7.69 ± 7.44	0.303	1.042
Twice or more	16.63 ± 15.30			5.90 ± 6.50		
**Which dentifrice does your child use for brushing his or her teeth**						
Toothpaste	16.52 ± 14.25	0.251	1.21	6.67 ± 6.89	0.195	-1.758
Toothpowder/Coal/Meswak	20.50 ± 8.05			2.33 ± 4.04		
**Rinsing the mouth after meals/sweets/fizzy drinks**						
Yes	19.30 ± 15.89	0.119	-1.574	6.67 ± 7.25	0.865	-0.17
No	14.89 ± 11.65			6.40 ± 6.48		
**Cleaning tongue regularly**						
Yes	16.36 ± 13.97	0.497	-0.685	6.74 ± 8.71	0.633	-0.481
No	18.48 ± 13.73			5.91 ± 4.89		

Independent sample t-test; bold number indicate *p* value < 0.05; SD: Standard deviation

The results of the multiple linear regression analysis of CPQ_8 − 10_ and OIDP scores are shown in Table 6. Thirteen variables were entered into both CPQ_8 − 10_ and OIDP total score models. After adjusting for confounding variables, caries status (β = 0.361 and *p* value = 0.001) and place of residence (β = 0.329 and *p* value = 0.032) were found to be strong and significant predictors of higher CPQ_8 − 10_ total score. The model is statistically significant (F = 2.365, *p* = 0.009) and the total exploratory effect in terms of adjusted R² was 0.149(14.9% variance in the CPQ_8 − 10_ total score after adjusting for the number of predictors). Additionally, gingivitis (β = 0.265 and *p* value = 0.035) maintained a statistical significant relationship with OIDP total score, indicating a significant positive effect on OIDP score. However, the model that considered OIDP total score as a dependent variable was not found to be statistically significant (F = 1.399, *p* = 0.180) and about 6.8% (Adjusted R²= 0.068) variance was explained by the model after adjusting for the number of predictors in the OIDP model. Other variables such as gender, age and education level of mother / institutional caregivers, type of caregivers, plaque and calculus status and oral health-related behaviors showed non-significant effects on both outcomes.

**Table 6:** Multiple linear regression analysis for the total CPQ_8 − 10_ and OIDP scores according to the sociodemographic characteristics and oral health status

Explanatory Variables	CPQ_8 − 10_ Total Score			*p* value	OIDP Total Score			*p* value
	Unstandardized Co-efficient		Standardized Coefficient		Unstandardized Co-efficient		Standardized Coefficient	
	B	SE	β		B	SE	β	
**Gender of children**	0.901	2.793	0.033	0.748	-1.104	1.868	-0.076	0.557
**Age of mothers/caregivers**	1.746	2.945	0.063	0.555	2.251	2.271	0.142	0.325
**Education of mothers/caregivers**	-0.413	3.232	-0.015	0.899	-0.953	1.895	-0.062	0.617
**Type of caregivers**	-2.339	4.527	-0.084	0.607	3.796	2.38	0.279	0.116
**Place of residence**	9.154	4.192	0.329	**0.032**	-4.697	3.511	-0.169	0.186
**Caries**	10.564	3.031	0.361	**0.001**	-5.842	7.16	-0.413	0.418
**Plaque**	-1.876	4.989	-0.066	0.708	-7.375	4.019	-0.417	0.071
**Calculus**	2.708	4.581	0.098	0.556	5.882	3.624	0.365	0.11
**Gingivitis**	5.524	3.15	0.182	0.083	3.65	1.694	0.265	**0.035**
**Model Summary**	F = 2.365;Adjusted R²=0.149; *p* = 0.009				F = 1.399;Adjusted R²=0.068; *p* = 0.180			

Multiple linear regression model; β = Standardized Coefficient, SE = Standard Error, bold number indicate *p* value < 0.05

## Discussion

This study represents the first effort to investigate the OHRQoL and its determinants among children of FSWs in Bangladesh which are associated with stigma, sexually transmitted diseases and human rights violations [36]. It also successfully employed previously validated OHRQoL questionnaires in a low-resource setting among children and adolescents, yielding good internal consistency reliability. Additionally, the study accounted for the multi-level influences on OHRQoL by adjusting for other potential confounding variables. The study also demonstrates how the children’s oral health was impacted by their mothers’ employment activities [37].

In terms of domain-specific scores, the average CPQ_8 − 10_ score in the children aged 7 to 11 was 16.92 ± 13.87, this finding is slightly higher than the mean total score in the same population in a previous study [38]. This age group experienced the greatest impact on oral symptoms and functional limitations. This finding aligns with another study conducted on the same age group and utilizing a similar assessment tool [38, 39]. However, reports from other population have displayed contrasting findings [40].

Adolescents aged 12 to 17 years in this study showed a higher prevalence of oral health impacts in the past three months, a rate notably higher than that reported among similar age groups in other Asian and European countries [41–43]. However, comparing these results to previous studies can be difficult due to cultural differences that may influence oral health outcomes [44, 45]. Eating and cleaning teeth were the most commonly affected activities, aligning with previous research in populations with similar cultural norms [46–48]. 

OHRQoL is particularly important for children and adolescents, as younger individuals are more sensitive to influences such as appearance. As children grow, their perceptions of health and quality of life change, impacting their current well-being, psychological development, social skills, and education [49]. Psychological effects related to oral health, like avoiding laughter or being teased about their teeth, were more prominent in younger individuals compared to adults and the elderly [50]. 

Gender disparity was observed across both age groups, with male sex identified as an independent non-significant risk factor for OHRQoL. This finding contrasts with previous studies, which reported that females had a higher overall impact score compared to males [43]. Children living in Dhaka city exhibited significantly better OHRQoL than those residing in the Daulatdia brothel. FSWs often lack time for their children due to their demanding work [9]. Organisations like Basha provide support by offering sustainable livelihoods, literacy courses, vocational training, and parenting classes to women who were previously trapped in the sex trade. The daycare facility associated with Basha offers education and extracurricular activities for their children [51, 52]. This holistic support may explain why children in institutional care have better OHRQoL compared to those living in brothels.

After considering other covariates, it was determined that dental caries positively and significantly influences oral well-being. A range of literature demonstrates that more severe dental caries negatively impacted children’s OHRQoL [39, 53–56]. Rather, OHRQoL, which demonstrated an improvement concerning functional limitation and emotional well-being, was positively associated with treated caries [57].

Adolescents with gingivitis showed significantly poorer OHRQoL than their counterparts. Conditions such as severe gingivitis, gingival bleeding (GB), and plaque formation are most likely negatively associated with children’s oral health and everyday life perspectives [58]. Furthermore, having signs of gingivitis raises the likelihood of continuing from attachment loss to tooth loss around 3.22-fold and 5.4-fold, respectively, when compared to non-bleeding locations [59]. Gingival inflammation may worsen in young people during puberty. High levels of sex hormones in the bloodstream have been found to be associated with gingival inflammation [60, 61]. OHRQoL pertains to the impact of oral health conditions on an individual’s daily functioning, overall health, and general quality of life.

From this study, it can be concluded that socio-behavioural and oral health factors significantly influence the OHRQoL of children. Factors such as parental education, socioeconomic status, and access to dental care contribute to children’s oral health outcomes and overall well-being. Additionally, oral health status and positive oral health knowledge, attitude, practices such as regular tooth brushing and healthy dietary choices, enhance OHRQoL by reducing the incidence of dental issues and promoting better self-esteem among children [60]. Policymakers must prioritize the development of oral health strategies tailored to enhance the well-being of marginalized and vulnerable populations, such as children of sex workers. The findings of this study provide valuable insights into the factors that predict OHRQoL in this demographic, highlighting the unique challenges they face in accessing care. By understanding these predictors, policymakers can design targeted interventions that address barriers to oral health services, ensuring these children receive the necessary support and treatment to improve their overall quality of life and foster greater accessibility to essential oral health services.

### Limitations

The study sample may not be fully representative of all children of sex workers in Bangladesh, as data were only collected from Dhaka City and Daulatdia. Accessing participants from other regions proved to be extremely challenging. Moreover, the study’s recruitment approach, which focused on one brothel and three shelter homes in Dhaka, may have introduced selection bias.

## Conclusion

The study demonstrates that children aged 7 to 11 in Daulatdia who exhibit caries have significantly lower OHRQoL. Furthermore, gingivitis is correlated with elevated OIDP scores in children aged 12 to 17. Consequently, integrating OHRQoL assessments into oral health surveys is essential for evaluating the effectiveness of preventive and therapeutic programs to enhance oral health outcomes. Furthermore, future interventional studies should be conducted to foster positive oral hygiene practices for the improvement of the quality of life for these marginalized individuals.

## Data Availability

No datasets were generated or analysed during the current study.

## Abbreviations

FSW: Female sex workers
NGO: Nongovernmental organization
OHRQoL: Oral health-related quality of life
CPQ_8 − 10_: Child perception questionnaire 8–10
OIDP: Oral impacts on daily performances
DMFT: Decayed, missing and filled teeth
GI: Gingival index
PI: Plaque index
CI: Calculus index
